# Chromosome-level Genome Assembly of the High-altitude Leopard (*Panthera pardus*) Sheds Light on Its Environmental Adaptation

**DOI:** 10.1093/gbe/evac128

**Published:** 2022-08-17

**Authors:** Chuang Zhou, Yi Liu, Rusong Zhang, Xiaofeng Zheng, Guangqing Zhao, Fengjun Li, Wei Liu, Bisong Yue, Nan Yang

**Affiliations:** Key Laboratory of Bioresources and Ecoenvironment (Ministry of Education), College of Life Sciences, Sichuan University, Chengdu, P.R. China; Key Laboratory of Bioresources and Ecoenvironment (Ministry of Education), College of Life Sciences, Sichuan University, Chengdu, P.R. China; Key Laboratory of Bioresources and Ecoenvironment (Ministry of Education), College of Life Sciences, Sichuan University, Chengdu, P.R. China; Key Laboratory of Bioresources and Ecoenvironment (Ministry of Education), College of Life Sciences, Sichuan University, Chengdu, P.R. China; Key Laboratory of Bioresources and Ecoenvironment (Ministry of Education), College of Life Sciences, Sichuan University, Chengdu, P.R. China; Key Laboratory of Bioresources and Ecoenvironment (Ministry of Education), College of Life Sciences, Sichuan University, Chengdu, P.R. China; College of Animal Science and Veterinary Medicine, Southwest Minzu University, Chengdu, P.R. China; Key Laboratory of Bioresources and Ecoenvironment (Ministry of Education), College of Life Sciences, Sichuan University, Chengdu, P.R. China; Institute of Qinghai-Tibetan Plateau, Southwest Minzu University, Chengdu, P.R. China; Collaborative Innovation Center for Ecological Animal Husbandry of Qinghai - Tibetan Plateau, Southwest Minzu University, Chengdu, P.R. China

**Keywords:** high-altitude leopard, chromosome-level genome, phylogenetic analysis, high-altitude adaptation, missense mutations

## Abstract

The leopard (*Panthera pardus*) has the largest natural distribution from low- to high-altitude areas of any wild felid species, but recent studies have revealed that leopards have disappeared from large areas, probably owing to poaching, a decline of prey species, and habitat degradation. Here, we reported the chromosome-scale genome assembly of the high-altitude leopard (HL) based on nanopore sequencing and high-throughput chromatin conformation capture (Hi-C) technology. *Panthera* genomes revealed similar repeat composition, and there was an appreciably conserved synteny between HL and the other two *Panthera* genomes. Divergence time analysis based on the whole genomes revealed that the HL and the low-altitude leopard differentiate from a common ancestor ∼2.2 Ma. Through comparative genomics analyses, we found molecular genetic signatures that may reflect high-altitude adaptation of the HL. Three HL-specific missense mutations were detected in two positively selected genes, that is, *ITGA7* (Ala112Gly, Asp113Val, and Gln115Pro) and *NOTCH2* (Ala2398Ser), which are likely to be associated with hypoxia adaptation. The chromosome-level genome of the HL provides valuable resources for the investigation of high-altitude adaptation and protection management of the vulnerable leopard.

**Significance**

The leopard (*Panthera pardus*), widely distributed from low- to high-altitude areas, is under substantial threats like illegal poaching, prey loss, habitat degradation, and human activities. In this study, we generated chromosome-level genome assembly from the high-altitude leopard and investigated its genetic mechanism of high-altitude adaptation. The newly generated data should contribute to a better understanding of the high-altitude adaptation and conservation of the vulnerable leopard.

## Introduction

Leopards (*Panthera pardus*) have pelage hues varying from pale yellow to deep golden and being patterned with black rosettes. They have the most expansive natural range of any large felid, occurring throughout sub-Saharan Africa, India, and southern Asia (Hayward et al. 2006), and their coat and color patterns vary widely across various types of habitats. The leopards inhabiting snowy temperate forests with low winter temperatures could display a pale cream-colored long-hair winter coat, which can possibly cause confusion with the snow leopard (*Panthera uncia*; Uphyrkina et al. 2001). Leopards can live at sea level, in foothill areas, in mountains, as well as in the Himalayas where they are sympatric with snow leopards up to 5,200 m. Leopards are currently listed as vulnerable by the International Union for the Conservation of Nature and in Appendix I of the Convention of International Trade in Endangered Species. Leopards are confronted with worldwide population declines as a result of substantial threats throughout its range like illegal poaching, prey loss, habitat degradation, and anthropogenic disturbances (Han et al. 2019). Besides protective legislation, habitat restoration, and reintroduction for conservation of endangered species, genomic approaches become more widely adopted in conservation. The reference genome is a key factor to investigate many biological problems that are crucial for species conservation, such as demography, inbreeding, hybridization, disease susceptibility, behavioral ecology, and adaptation. For example, the reference genome of the Tasmanian devil (*Sarcophilus harrisii*), an endangered Australian marsupial, played an indispensable role in understanding an infectious clonal cancer devil facial tumor disease and the management of the species in the wild. Therefore, a high-quality reference genome is an important conservation and management tool for the protection and long-term survival of the vulnerable leopard.

High-altitude leopards (HLs) primarily live in the mountain ranges where they are sympatric with snow leopards, and there are severe physiological challenges such as low oxygen level, high ultraviolet (UV) radiation, aridity, and low primary productivity (Verma et al. 2012; Janecka et al. 2017). Advantageous genetic mutations and selective pressure were considered as the contributing factors for adaptation to a high-altitude environment. Many studies have sought to determine the molecular genetic basis for the successful adaptation of high-altitude animals like yak (Qiu et al. 2012), gray wolf (Zhang et al. 2014), Tibetan antelope (Ge et al. 2013), goat (Song et al. 2016), and chicken (Wang et al. 2015). Some responsible genes have been worked out by previous studies, among which *EPAS1* (endothelial PAS domain protein 1) and *EGLN1* (egl-9 family hypoxia-inducible factor [HIF] 1) have been the most prominent ones. *EPAS1* and *EGLN1* are key genes associated with the HIF pathway and possess functional mutations (Lorenzo et al. 2014; Xu et al. 2014). Convergent evolution has occurred in distantly related organisms under the same selective pressures to adapt to a high-altitude environment. For example, the *EPAS1* gene was reported to be one vital influencing factor in high-altitude adaptation, which was shared by the snow leopard (Cho et al. 2013), Tibetans (Simonson et al. 2010; Peng et al. 2011; Wang et al. 2011), Tibetan gray wolf (Zhang et al. 2014), Tibetan mastiff (Gou et al. 2014; Li et al. 2014; Wang et al. 2014), and Tibetan goat (Song et al. 2016). On the contrary, different geographic populations of the same species could adapt to high-altitude conditions through different genes or functional pathways, such as human (from Tibet, The Andes, and Ethiopia; Bigham et al. 2010; Simonson et al. 2010; Scheinfeldt et al. 2012) and Tibetan pig (from Tibet, Gansu, Sichuan, and Yunnan province in China; Ai et al. 2014). The genetic mechanism of high-altitude adaptation in the HL, however, remains perplexing. Therefore, understanding genetic factors that underlie adaptation to high-altitude conditions could fill important gaps in our use and understanding of conservation genetics to support HL conservation.

Currently, most of reference genomes were assembled based on short reads generated through second-generation sequencing technologies (such as Illumina or Roche 454) with limited contiguity and quality. Compared with second-generation sequencing technologies, long-read sequencing technologies (such as Oxford nanopore and PacBio SMRT) can generate long reads and enables the assembly of a genome with a high level of completeness. These long reads can span complex regions that have not been sequenced. The value of the ultra-long reads produced by Oxford Nanopore Technologies (ONT) has been reported in improving the contiguity and completeness of assembled genomes (Ge et al. 2019). In addition, high-throughput chromatin conformation capture (Hi-C) technology allows the genome sequences to be assembled to the scale of full chromosomes based on capture of all DNA interaction patterns in chromatin (Lieberman-Aiden et al. 2009). In this study, we combined nanopore long-read sequencing and Hi-C sequencing to generate a high-quality genome for the HL. This chromosome-scale genome can lay a strong foundation for fully understanding the diversity and population dynamics of the HL and can provide a sound support for breeding, functional genomic research, and species conservation.

## Results and Discussion

### Genome Sequencing and Assembly

The detailed sequencing data are summarized in supplementary table 1, Supplementary Material online. In total, 694.64 Gb of sequencing data was generated, of which 164.16 Gb (67.19 × coverage) was produced from Illumina reads, 264.53 Gb (98.52 × coverage) from ONT reads and 265.95 Gb (108.77 × coverage) from the Hi-C library. A k-mer depth of 50 was the highest peak in the figure, and the k-mer number 123,373,857,631 was used to calculate the HL genome size (supplementary fig. 1, Supplementary Material online). The genome size of the HL was estimated to be 2.44 Gb, and the heterozygosity was about 0.20%. The repeatability was about 52.81% and the GC content was about 42.58%. The genome was assembled with ONT long reads and yielded 2.43 Gb of reference genome size with contig N50 length about 60.11 Mb and a longest length of 166.45 Mb. In the case of the Hi-C super-scaffolding, the total size of the genome was 2.43 Gb with the N50 value of 147.15 Mb in length. The HL genome was the best assembled in the *Panthera* taxon (table 1). Although the N50 scaffold of the lion was as high as 136.05 Mb, its N50 contig (0.29 Mb) was far lower than that of the HL (60.11 Mb). The genome size of the final assembled version is almost the same as that estimated by k-mer, indicating the accuracy of the assembly. In the *Panthera* that have been sequenced, the HL genome (2.43 Gb) was larger than the *Panthera tigris* (tiger; 2.39 Gb) and *Panthera leo* (lion; 2.41 Gb), smaller than the *Panthera onca* (jaguar; 2.50 Gb) and low-altitude leopard (LL; 2.58 Gb).

**Table 1:** Assembly Statistics of the HL and Other *Panthera* Species

Species	HL	*P. tigris*	*P. leo*	*P. onca*	LL
Sequencing technology	Illumina; Oxford Nanopore; Hi-C	Illumina	Illumina; Oxford Nanopore; 10X Genomics	Illumina	Illumina
Assembly size (Gb)	2.43	2.39	2.41	2.51	2.58
Number of scaffolds	3,384	1,479	8,060	438,792	50,377
N50 scaffold (Mb)	147.15	8.86	136.05	0.12	21.70
Number of contigs	4,607	157,032	23,775	460,121	265,330
N50 contig (Mb)	60.11	0.03	0.29	0.06	0.02
GC%	41.7%	41.5%	41.6%	41.7%	41.9%

Note. The assembly statistics of other *Panthera* genomes were taken from the NCBI assembly database. The GenBank assembly accession numbers were as follows: *P. tigris* (GCA_000464555.1), *P. leo* (GCA_008795835.1), *P. onca* (GCA_004023850.1), and LL (GCA_001857705.1).

**Fig. 1.**
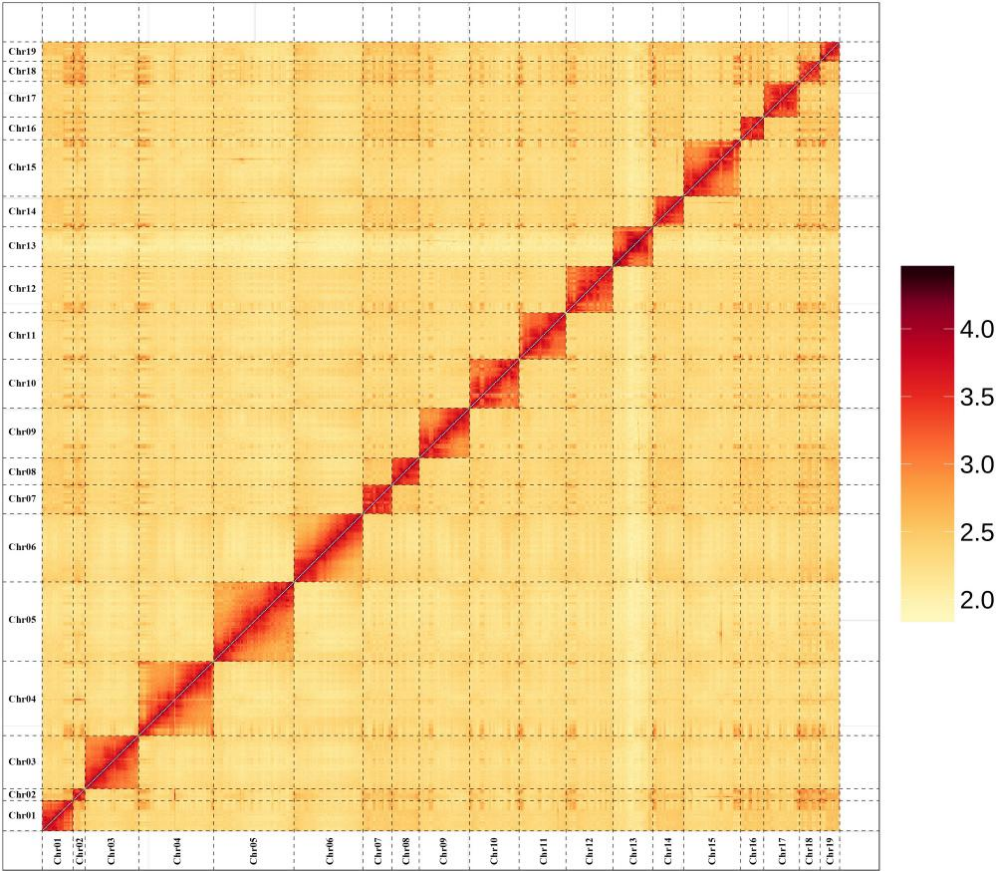
Hi-C interaction heat map between 19 chromosomes for the HL genome.

To assess the quality of chromosome-level genome assembly, a Hi-C heat map of the whole genome visualized it. There were 19 chromosomes in the figure, and the interaction signal strength of the two pairs of chromosomes around the diagonal was much stronger than the interaction strength of the chromosomes at other positions (fig. 1), which indicate that the quality of the genome assembly was very high. We evaluated the completeness of the complete genome assembly through calculating coverage for a set of single-copy orthologous genes in mammals using Benchmarking Universal Single-Copy Orthologs (BUSCO), which revealed a genome coverage rate of 94.8%. Although the BUSCO values of near-source species were not much different: 96.1% for lion, 95.5% for LL, and 94.3% for tiger, the BUSCO value of jaguar was 81.9% due to the higher proportion of fragmented BUSCOs (12.3%) than that of other species (supplementary fig. 2, Supplementary Material online). The lower the fragmented BUSCOs, the better the assembly quality. The possible reason is that although the fragmented gene reached the predicted score, the length did not meet the requirement. It is possible that the fragment was not assembled during the genome assembly process, or it may be that the gene was not completely predicted due to the particularity of the gene structure during gene prediction. In order to evaluate the accuracy of assembly, the comparison rate of Illumina library reads to the assembled genome was about 98.84%, and the coverage rate was about 97.90%, indicating that the reads and the assembled genome have good consistency. There are a total of 1,104,323 SNPs in the HL genome (heterozygous SNPs 1,098,046, homozygous SNPs 6,277), accounting for 0.047% of the total genome.

**Fig. 2.**
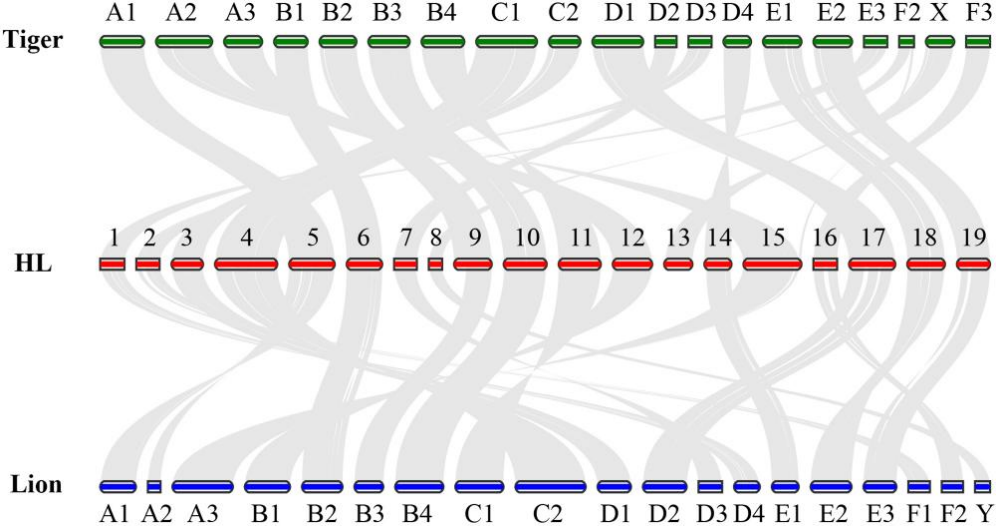
Examples of chromosome rearrangements. The lines between the two horizontal lines link the alignment blocks.

**Fig. 3.**
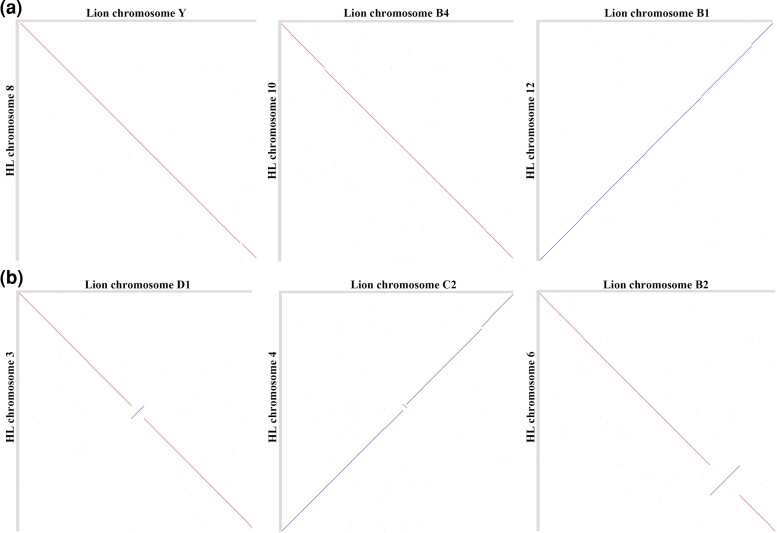
Alignments of the HL chromosomes to lion chromosomes. (*a*) Examples of high synteny and high assembly correctness of HL chromosomes. (*b*) Examples of chromosomal rearrangements between the HL and lion. Forward alignments are in blue, and reverse alignments are in red.

Given the close relationship between HL and the other two *Panthera* species and the size and quality of the draft genome for the other two *Panthera* species, we performed genomic synteny analyses. Substantial genome-wide colinearity was illustrated between HL and other two *Panthera* species (fig. 2). For deeper understanding of chromosomal synteny, we took the HL and lion genomes, for example, to conduct one-to-one chromosomal alignment. The findings showed close overall genome synteny between HL and lion. The X, B4, and B1 chromosomes of the lion and the HL chromosomes 8, 10, and 12 have very good collinearity, which can be said to be completely coincident (fig. 3*a*). In addition, chromosomes D1, C2, and B2 of the lion and chromosomes 3, 4, and 6 of the HL showed a high degree of coincidence in addition to a small number of rearrangements (fig. 3*b*). Differences in genomic structure between closely related species are considered to be a major factor in species diversification, because gene requires recombination in collinear chromosomes (Zhang et al. 2004).

### Genome Characterization

**Fig. 4.**
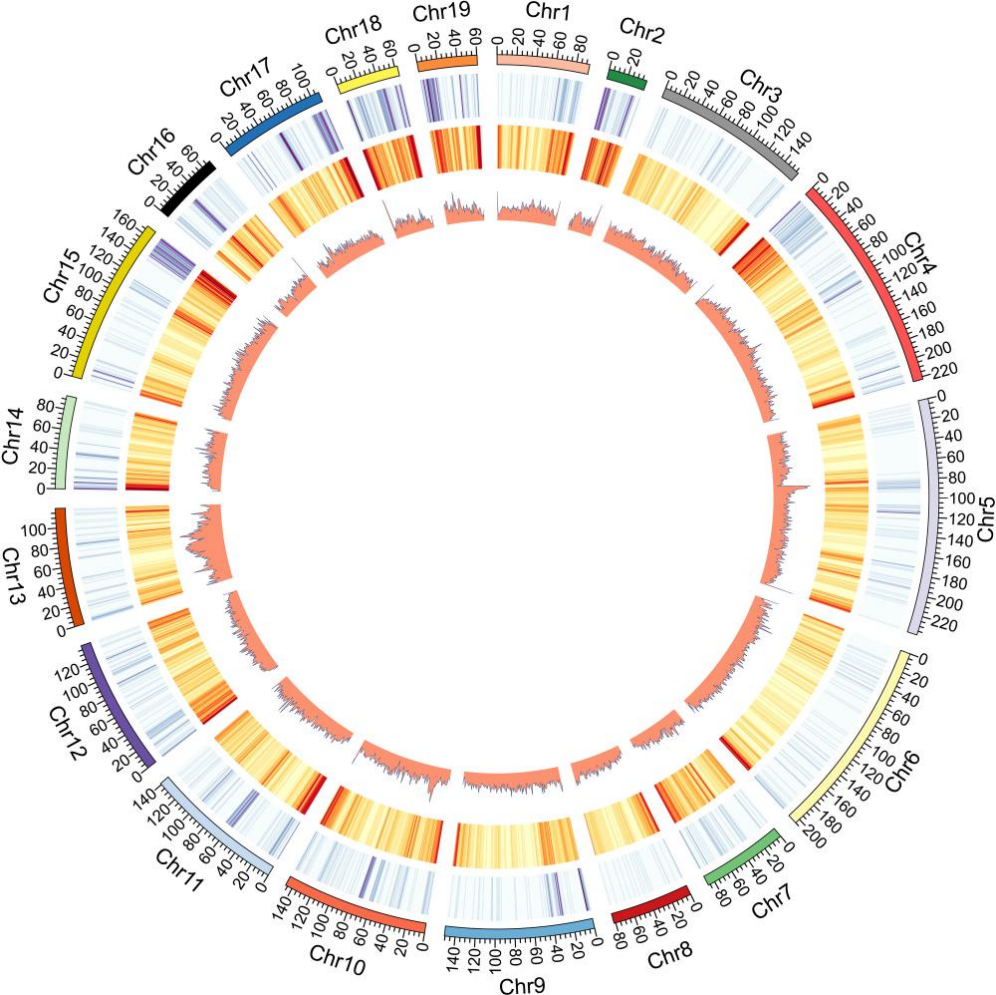
The genome landscape of HL. From outer to inner circles: the 19 chromosomes at the Mb scale, gene density, GC density, and repeat density across the genome, respectively, drawn in 1 Mb nonoverlapping windows.

We found that 36.97% of the HL genome was composed of repetitive elements, including long interspersed elements (22.68%), short-terminal repeat (6.93%), long-terminal repeat (5.27%), and DNA elements (3.22%) (supplementary table 2, Supplementary Material online). A total of 1,323,721 perfect simple sequence repeats (SSRs) were identified, including 494,352 mono-, 518,799 di-, 58,490 tri-, 193,811 tetra-, 44,212 penta-, and 14,057 hexa-nucleotide SSRs (supplementary table 3, Supplementary Material online). The obtained consensus gene set composed of a total of 19,120 protein-coding genes (PCGs), lower than that of Amur tiger (20,226). The longest chromosome of the HL was chromosome 5 (238.91 Mb), and the chromosome with the most coding genes was chromosome 2 (9,972), which is far more than other chromosomes (table 2). Homology- and structure-based strategies were employed for functional annotation of PCGs. We found functional annotation for 19,102 PCGs, which represents about 99.91% of all the genes (supplementary table 4, Supplementary Material online). For clarity, the distributions of gene density, GC density and repeat density across the 19 HL chromosomes were further illustrated (fig. 4). Generally, the regions with low gene density had high repeat content, and the regions with high repeat content usually had high GC content.

**Table 2:** The Statistics and Characteristics of the HL Chromosomes

Chromosome	Chromosome size (Mb)	Anchored scaffold number	Anchored gene number	Percentage of repetitive sequences (%)	GC content (%)
Chr1	93.18	50	672	36.83	43.08
Chr2	39.30	42	643	37.00	48.49
Chr3	157.78	15	877	39.45	39.65
Chr4	222.61	103	1,730	38.01	41.46
Chr5	238.91	129	1,265	38.43	39.80
Chr6	205.14	59	987	40.24	39.04
Chr7	86.92	4	630	36.99	43.27
Chr8	81.73	100	370	38.25	40.37
Chr9	150.65	79	1,047	39.13	40.15
Chr10	147.15	74	1,194	39.94	41.87
Chr11	141.31	55	1,172	39.00	41.31
Chr12	139.24	44	1,096	37.61	42.89
Chr13	119.75	130	785	53.80	40.62
Chr14	93.56	66	753	37.65	42.60
Chr15	168.59	68	1,613	37.26	42.27
Chr16	69.36	73	657	37.40	42.77
Chr17	108.80	11	1,286	38.89	42.53
Chr18	62.86	116	1,047	33.97	47.50
Chr19	60.34	40	920	37.84	46.17
Sum	2,387.18	1,258	18,744		

### Gene Family, Phylogeny, and Divergence

**Fig. 5.**
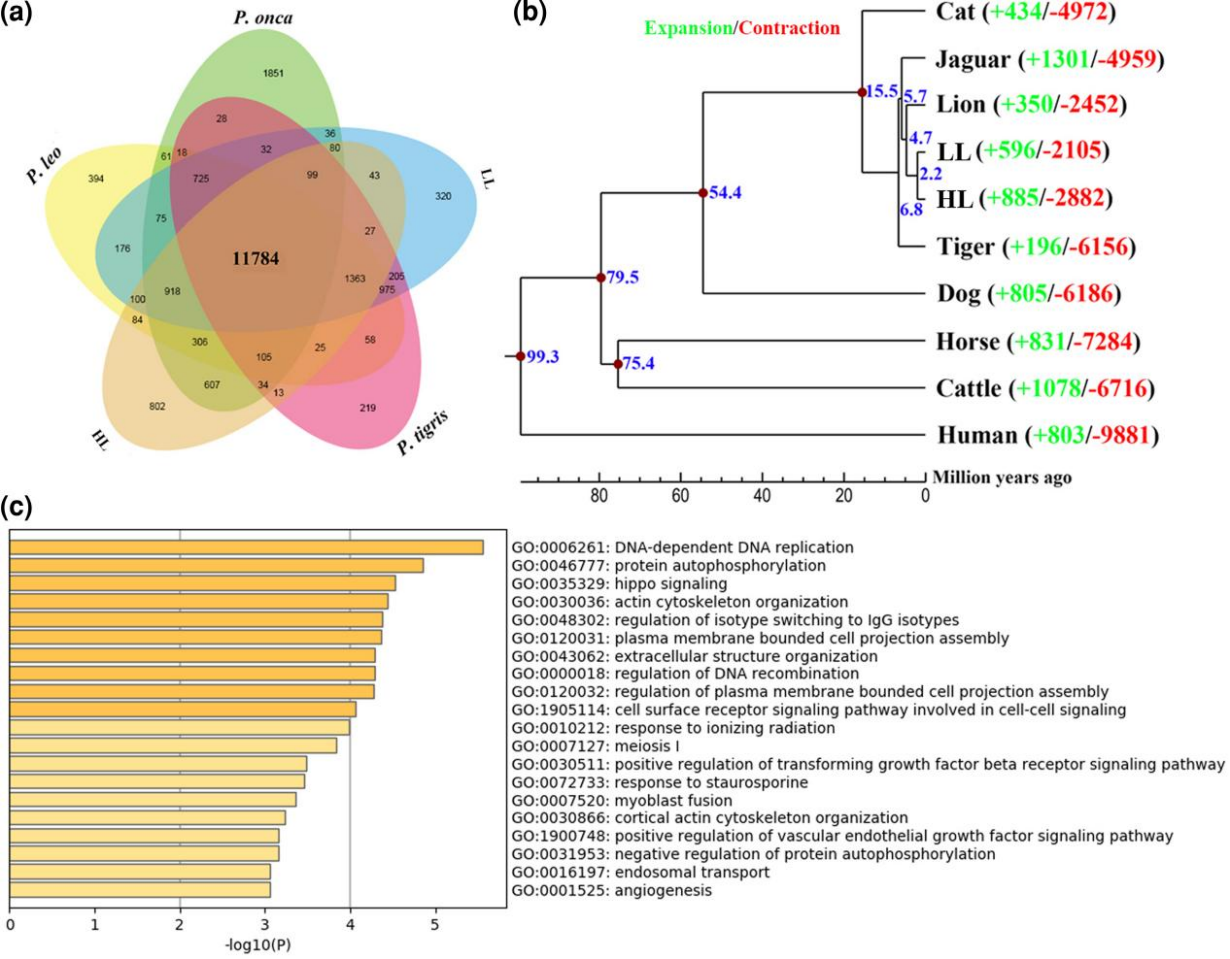
Comparative genomics analysis related to the HL. (*a*) Orthologous gene clusters among *Panthera* species. (*b*) Phylogenetic tree constructed using one-to-one orthologous genes. The time lines indicate divergence times among the species. (*c*) Enrichment analysis of the PSGs of the HL.

A gene family, a group of homologous genes with similar structures, generally has similar functions (Demuth et al. 2006). A total of 19,120 HL genes were clustered into 15,709 gene families with an average of 1.22 genes per gene family. We identified 51 HL-specific gene families which have 154 HL genes among the ten mammalian species included in the analysis. Furthermore, there were 802 HL-specific gene families among *Panthera* species (fig. 5*a*). These lineage-specific gene families possibly have contributed to the evolution of the HL. The phylogenetic tree constructed using single-copy orthologs revealed that leopard was most closely clustered with lion, and further, formed into a clade genus *Panthera* (fig. 5*b*), which was in line with previous studies (Christiansen 2008; Bagatharia et al. 2013). The HL diverged approximately 2.2 Ma from its common ancestor and the genus *Panthera* diverged about 15.5 Ma from the genus *Felis*.

In order to examine the evolutionary history of gene families, the expansion and contraction in the HL genome were estimated in comparison with other mammals. We found that the HL genome composed of 885 expanded gene families and 2,882 contracted gene families (fig. 5*b*). The 885 HL expanded gene families contained 1,246 genes, whereas 2,249 genes for 2,882 contracted gene families. In this study, it was observed that many expanded gene families were distributed in pathways associated with high-altitude adaptation in the HL genome such as HIF (GO:0102113), response to hypoxia (GO:0001666), and response to UV (GO:0009411). The study of nucleotide polymorphisms in Tibetans reveals strong evidence that *HIF2α* and other genes in the HIF signaling pathway were positively selected (Rankin et al. 2007). In addition to hypoxia, strong UV radiation environments in high-altitude areas are also the main limiting factor for the successful colonization of animals. Over the higher altitude of Tibetan Plateau, the UV erythemal dose has a higher value with the multi-yearly mean value is about 5,500 J·m^–2^, and over some regions, the value is up to 6,000 J·m^–2^, whereas the low-altitude area is only 1,500 J·m^–2^ (Xiao and Jiang 2013). Many significantly contracted gene families in the HL were distributed in olfactory receptor activity (GO:0004984) and immune response (GO:0006955). Due to the thin air, single environment, and simple odor molecules in the air at high altitudes, the olfactory receptor genes of species usually shrink significantly compared with lower altitudes (Li et al. 2013). The whole-genome sequencing of Tibetan chickens and ground tits living on the plateau showed a large number of olfactory receptor genes were lost in the genome, thereby reduced energy consumption during olfactory perception (Qu et al. 2013; Wang et al. 2015). In addition, the high-altitude environment has strong UV radiation and fewer types of pathogenic microorganisms, so a lot of immune-related genes have contracted or lost at high altitudes. However, the immune genes have adaptively evolved in Tibetan chickens may be related to artificial domestication pressure was greater than natural selection pressure (Zhang et al. 2016).

### Positive Selection

Positive selection provides evolutionary innovation in specific adaptation, and the positively selected genes (PSGs) in HL had a significantly higher ratio of nonsynonymous substitutions to synonymous substitutions than other genes. Based on orthologous gene annotation by OrthoFinder2 across ten mammals, 6,463 single-copy genes were used for positive selection analyses. As a result, we identified 817 positive selected genes in the HL using the branch-site model in PAML. The gene ontology (GO) and KEGG enrichment analyses showed significant terms and pathways were involved in high-altitude adaptation, such as vascular smooth muscle contraction (KEGG map04270), positive regulation of vascular endothelial growth factor signaling pathway (GO:1900748), angiogenesis (GO:0001525), regulation of DNA recombination (GO:0000018), and response to ionizing radiation (GO:0010212) (fig. 5*c*). Hypoxia has a greater impact on the animal’s cardiovascular system, and high-altitude species mainly improve hypoxic tolerance by enhancing myocardial contraction and angiogenesis. In the ground tit, PSGs (*HIF1AN*, HIF 1 subunit alpha inhibitor; *ANGP*, angiopoietin; *ADAM* family, etc.) were involved in angiogenesis, cardiopulmonary development, and reactive oxygen metabolism (Wang et al. 2015). These genes also existed in high-altitude yaks and humans, indicating different animal groups have similar adaptation mechanisms to hypoxic response (Simonson et al. 2010; Qiu et al. 2012). *VEGF* is the main factor that induces angiogenesis in vivo. Studies on *VEGF* in yak lung blood vessels have found that *VEGF* was scattered only on the alveolar wall. Under normal oxygen concentration, the expression of *VEGF* was very low, which played a role in maintaining the balance of blood vessel density. On the contrary, the expression increased significantly during hypoxia, because vascular endothelial cells secreted some growth factors to control the proliferation and growth of smooth muscle cells and fibroblasts, making the structure of yak lungs different from cattle at low altitudes. Through comparative genomics analysis, eight common amino acid substitutions of six genes (*RNASE4*, *DNAH11*, *CDT1*, *RTEL1*, *ARMC2*, and *NT5DC1*) were found in three high-altitude golden monkey species, which were related to lung function, DNA repair, and angiogenesis. Ultraviolet irradiation experiments were carried out on the *CDT1* gene related to DNA repair. The results showed that the mutant *CDT1* (A537V) has stronger stability than the wild type. It is speculated that the mutation will help the golden monkey to resist UV rays in a high-altitude environment. Detection of the *RNASE4* gene related to angiogenesis found that the mutant *RNASE4* (N89K + T128I) has higher activity in inducing HUVEC cells to generate tubular structures. It is speculated that these two mutations may enhance the angiogenic ability of *RNASE4* and help the golden monkey adapt high-altitude environment (Yu et al. 2016).

**Fig. 6.**
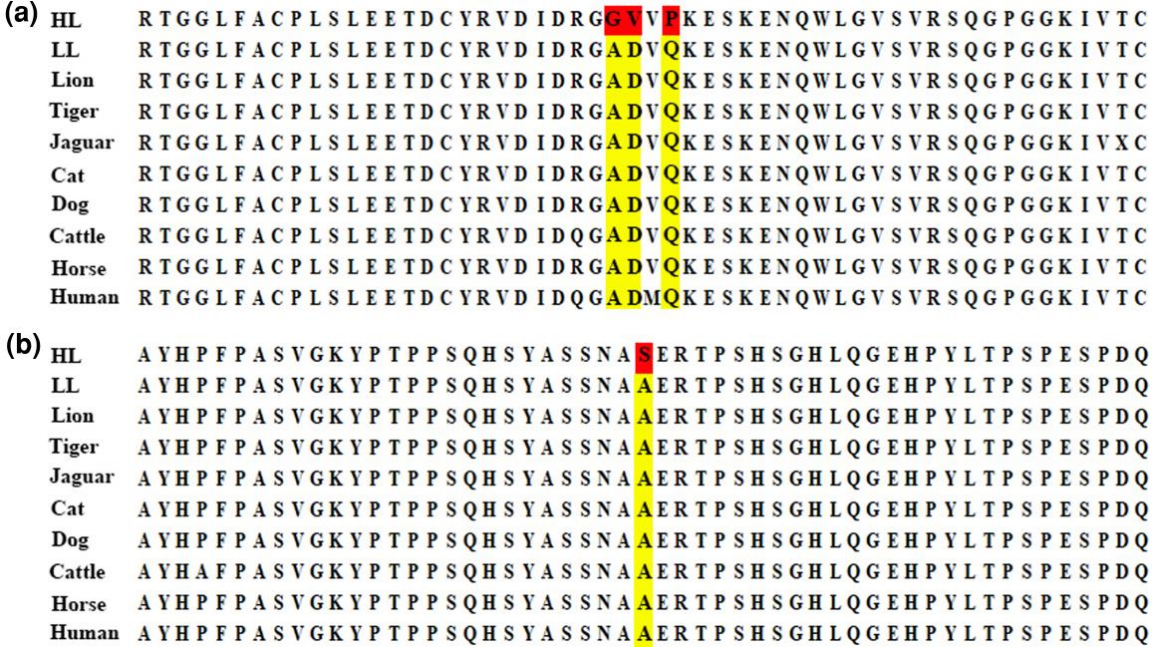
Missense mutations in the HL. (*a*) Amino acid sequence alignment of *ITGA7*. The HL-specific missense mutations in *ITGA7* are marked in red. (*b*) Amino acid sequence alignment of *NOTCH2*. The HL-specific missense mutation in *NOTCH2* is marked in red.

Further examination of these 817 PSGs found that two genes (*ITGA7* and *NOTCH2*) functionally associated with hypoxia adaptation have four HL-specific missense mutations, that is, *ITGA7* (Ala112Gly, Asp113Val, and Gln115Pro) and *NOTCH2* (Ala2398Ser; fig. 6), which suggested a role in the high-altitude adaptation of the HL. *ITGA7* participates in PI3K-akt and MAPK signaling pathways, indirectly regulates VEGF and HIF signaling pathways and enhances hypoxic adaptation (Zhang et al. 2015). Notch signal directly or indirectly participates in the regulation of angiogenesis through interactive dialogue with vascular endothelial growth factor, BMP-SMAD signaling pathway, extracellular matrix molecules, etc., and sustained Notch signal is to maintain the stability of the structure and function of adult vascular system. *NOTCH1* loss of function can cause zebrafish vascular remodeling disorders, including the collapse of the dorsal large artery (Fish and Wythe 2015). High concentration of vascular endothelial growth factor can induce the expression of arterial marker genes ephrinB2, *Dll4* and *NOTCH4* (Zhang et al. 2008).

## Conclusions

Here we assembled the chromosome-level genome of the HL by de novo assembly of long reads produced by nanopore sequencing and Hi-C. The reference quality genome (2.43 Gb) of the HL has the assembled contig N50 of 60.11 Mb and the longest contig of 166.45 Mb. By utilizing Hi-C technology, we assembled contigs into scaffolds that resulted in a chromosome-level genome assembly with 19 chromosomes and a scaffold N50 length of 46.03 Mb. The genome was functionally annotated to produce a total of 19,102 (99.91%) PCGs. The phylogenetic analysis showed that the leopard was most closely related to the lion in *Panthera* species, and the HL diverged from the common ancestor 2.2 Ma. The functional enrichment analysis of PSGs in the HL revealed significant terms and pathways which were associated with high-altitude adaptation. We reported four HL-specific missense mutations detected in two PSGs, that is, *ITGA7* (Ala112Gly, Asp113Val, and Gln115Pro) and *NOTCH2* (Ala2398Ser), which possibly played a pivotal role in the hypoxia adaptation of HL. The chromosome-scale genome of the HL provides an invaluable resource for fully understanding the diversity and population dynamics of the HL and can provide a sound support for breeding, functional genomic research, and species conservation of the leopard.

## Materials and Methods

### Sample Collection and Sequencing

The muscle sample preserved in the Natural Museum of Sichuan University was collected from a naturally dead male HL in Baiyu county, Sichuan province where the altitude was almost above 3,500 m. Sodium dodecyl sulfate extraction method (50 ml) was used to standardize the operation procedure to extract DNA. Agarose gel electrophoresis was used to analyze the purity and integrity of DNA. Nanodrop detected the purity of DNA (OD260/280 ratio). Qubit fluorimeter (Invitrogen, Carlsbad, CA, USA) accurately quantified the DNA concentration. Second-generation sequencing was performed on Illumina NovaSeq PE150. The DNA samples passed the sequencing test were randomly broken into fragments with a length of 350 bp by the Covaris breaker. NEB Next® Ultra DNA Library Prep Kit (NEB, Ipswich, MA, USA) was used for library construction, and the reagents and consumables recommended in the instructions were strictly used.

ONT sequencing was conducted on flow cells of a PromethION 24 sequencer (version R9.4.1, ONT, UK). First use BluePippin (Sage Science, Beverly, MA, USA) to select DNA size selection (30–80 kb). The nanopore library was then constructed using the Ligation Sequencing Kit 1D (SQK-LSK109; New England Biolabs). It mainly includes three steps: (1) use NEBNext End repair/dA-tailing Module (E7546; New England Biolabs) for DNA repair and NEBNext FFPE DNA Repair Mix (M6630) for end-prep; (2) use NEBNext Quick Ligation Module (E6056; New England Biolabs) for Adapter ligation and clean-up; and (3) priming and loading the flow cell.

The Hi-C library was sequenced on Illumina NovaSeq PE150. The tissue was treated with paraformaldehyde, a cell cross-linking agent, to fix the chromatin conformation in the nucleus. After cell lysis, the above-mentioned cross-linked fixed chromatin was treated with restriction enzyme HindIII to create gaps on both sides of the cross-linking point. When the end was repaired, the biotin-14-dATP was added to label the end of the oligonucleotide. Nucleic acid ligase joined adjacent DNA fragments. The protease digested the protein at the junction to release the cross-linking state of the protein and DNA. Covaris M220 (Covaris, Woburn, MA, USA) was used to randomly break the extracted genomic DNA into 350 bp fragments. Under the adsorption of avidin magnetic beads, the DNA with biotin is captured, and the entire library preparation was completed in strict accordance with the steps of terminal repair, addition of A, linker connection, polymerase chain reaction amplification, and purification of the DNA fragments.

### Genome Assembly

Illumina data were used to estimate the genome size of the HL, and k-mers were counted by jellyfish version 2.2.9 (Marçais and Kingsford 2011) with 17-base oligonucleotide. The depth of ONT data coverage was calculated according to the estimated genome of survey. Then nanoplot version 1.18.2 was employed for quality control of nanopore data. Finally, we used wtdbg version 1.2.8 (Ruan and Li 2020) to preassemble nanopore data to obtain genome contig sequence (parameter -p 19 -k 0). Two rounds of polishing were performed on the contigs obtained from the initial assembly. Based on the default parameters of Racon version 1.32, nanopore data were used to polish the contig sequences (Vaser et al. 2017), and according to pilon version 1.22 (Walker et al. 2014), Illumina data were used to polish the contig sequences (parameter: -Xmx30g -diploid -changes -threads 8). In order to raise the assembled contigs to the chromosomal level, the ALLHiC (Zhang et al. 2019) was used to uniquely map Hi-C reads into contigs, and to retain the 500 bp flanking region of the restriction site (MboI) for further analysis. The number of chromosomes was 19 and the restriction sites were 50. In addition, low-quality matches were filtered according to the pipeline (https://github.com/tangerzhang/ALLHiC/wiki). Subsequently, the genetic algorithm optimized in ALLHiC was used to order and orient the contigs. In order to assess the completeness and accuracy of genome assembly, BUSCO version 2.0 (Simão et al. 2015) evaluated the quality of the genome. Furthermore, we selected Illumina library reads using BWA-MEM v.0.7.17 (Li and Durbin 2009) to compare with the assembled genome, counted the comparison rate of reads, the extent of genome coverage and the distribution of depth, and evaluated the integrity of the assembly and the uniformity of sequencing. Then we used samtools v1.3.1 (Li et al. 2009) to sort the results of BWA by chromosome coordinates, removed duplicate reads, performed SNP Calling, filtered the original results, and finally got the SNP statistical results.

### Genome Synteny Analysis

To visualize the concordance between the HL and other two chromosome-level *Panthera* genomes, we conducted genomic synteny analyses with MCscan (Python version; Tang et al. 2008) and LAST (Kiełbasa et al. 2011) v746.

### Characterization of Repeats

There are a large number of repeat elements in the nuclear DNA in most eukaryotic genomes, which have been indicated to have structural and functional roles (Biscotti et al. 2015). Repeatmodeler v1.0.3 (Benson 1999), including RECON v1.08 (Bao and Eddy 2002) and repeatscout v1.0.5 (Price et al. 2005), was employed to construct a de novo repeat library based on the complete genome with default parameters. Then the repeat consensus database with classification information (Tarailo-Graovac and Chen 2009) was merged with the Repbase (Jurka et al. 2005) database to construct a repeat consensus database. Repeatmasker v4.0.6 (Tarailo-Graovac and Chen 2009) was adopted to predict interspersed repeat elements in the whole genome against the Repbase and de novo repeat libraries with default parameters. Krait tool (Du et al. 2018) was employed to predict and characterize genome-wide microsatellite (SSR) loci of the whole genome, which can identify the loci that could be used for population genetic studies.

### Gene Prediction and Annotation

Using the repeat-masked genome, we combined de novo and homology-based approaches to predict gene models. In the de novo method, the software packages GENSCAN v3.1 (Burge and Karlin 1997), AUGUSTUS v2.4 (Stanke and Waack 2003), and GLIMMERHMM v3.0.4 (Majoros et al. 2004) were employed for predicting the PCGs of the HL genome with internal gene models. For the homology-based prediction, protein sequences from six mammals (lion, LL, tiger, cat, dog, and human) were aligned to the HL genome with TBLASTN. High-scoring segment pairs were concatenated using solar (version 0.9.6). GENEWISE (version 2.2.0; Birney et al. 2004) was used to analyze these alignments to determine accurately spliced alignments. Finally, EVidenceModeler v1.1.1 was used to integrate the above evidences with different weights for each to generate a consensus gene set (Haas et al. 2008). To functionally annotate the predicted genes of the HL, their protein sequences were used as queries to search against the swissprot and trembl protein databases (Bairoch and Apweiler 2000) using BLASTP with an *E*-value cutoff of 1 × 10^−5^. In order to annotate the functional motifs and protein domains, we employed the InterproScan tool (version 5.27; Hunter et al. 2009) in coordination with publicly available databases including Gene3D, PRINTS, Pfam, CDD, SMART, MobiDBLite, and PROSITE. GO IDs for each gene were assigned based on the results of SwissProt. To find the best match and the involved pathway for each gene, all genes were uploaded to KAAS (Moriya et al. 2007), a Web server for functional annotation of genes against the manually corrected KEGG gene database by BLAST, using the bidirectional best hit method.

### Gene Family Identification and Phylogenetic Analysis

Orthologous gene clusters and 1:1 orthologous gene sets among ten mammals (HL, LL, lion, tiger, jaguar, cat, dog, cattle, horse, and human) were identified using the package Orthofinder2 (Emms and Kelly 2018) with diamond as a protein aligner (Buchfink et al. 2015). The software prank v3.8.31 was used to align the sequences of 1:1 orthologous genes from these ten mammals. The alignment outputs were treated with Gblocks (Castresana 2000) to remove poorly aligned sequences. Then, the coding sequences of 1:1 orthologous genes were concatenated for each species to develop a super sequence for building the tree. The maximum likelihood phylogenetic tree was constructed using RAxML (Stamatakis 2014) with the GTRGAMMA model and 1,000 rapid bootstrap replicates. Human was set as the outgroup. McMctree as implemented in the PAML package (Yang 1997) was used to predict divergence times. Five calibration times were obtained from the TimeTree database (http://www.timetree.org/), namely, divergence times of cat and lion (12.2–16.6 Ma), cat and dog (51.0–56.0 Ma), cat and horse (70.2–79.0 Ma), cat and human (91.0–101.0 Ma), and cattle and horse (76.0–82.0 Ma). Café tool (version 4.0) was used to analyze the expansion and contraction of orthologous gene families between the ancestor and each of the ten species (Han et al. 2013). The gene family results from Orthofinder2 (Emms and Kelly 2018) and a tree with estimated divergence times between species were used as inputs. We used a criterion of *P* < 0.05 for significantly changed gene families, and the separate birth (*λ*) and death (*μ*) rates were estimated with the same program using the lambda/mu command with –s and –t options.

### Positive Selection Analysis

The above one-to-one gene clusters and the phylogenetic tree between the ten mammals were used to identify PSGs. The lineage HL was designated as “foreground” phylogeny. The software prank was used to align the coding sequences, and poorly aligned sequences with gaps were removed with a codon model using Gblocks. The values of d*N*, d*S*, and *ω* were estimated with the codeml program implemented within the PAML package (Yang 2007). The basic and branch-site models were tested, and genes under relaxation of selective pressure were eliminated by Likelihood ratio tests. We then identified the PSGs of the HL by means of FDR adjustment with *Q* values <0.05. The functional categories and pathways enriched in the PSGs were analyzed by using Metascape (Zhou et al. 2019). The HL-specific missense mutations were validated by comparing them with more mammals with publicly available genomes.

## Supplementary Material

evac128_Supplementary_Data

## Data Availability

The high-altitude leopard whole-genome sequencing data and the chromosome-level genome assembly have been deposited to the CNSA (CNGB Nucleotide Sequence Archive) with accession CNP0001205 (https://db.cngb.org/cnsa/), and the NCBI under the accessions SRR13500268, SRR13500269, SRR13500270, SRR13500271, SRR13500272, SRR13500273, SRR13500274, SRR13500275, SRR13500276, and SRR13500277.
