# Supplementary material for: Chromosome-level Genome Assembly of the High-altitude Leopard (Panthera pardus) Sheds Light on Its Environmental Adaptation
Source: Genome Biol Evol. 2022 Aug 17;14(9):evac128. doi: 10.1093/gbe/evac128 (PMC9452791; doi:10.1093/gbe/evac128)
Supplement: evac128_Supplementary_Data [file evac128_supplementary_data.zip › Supplementary_material_.docx]

**Table S1** Sequencing information of the HL.

| **Pair-end libraries** | **Insert size** | **Read length (bp)** | **Raw data (G)** | **Clean data (G)** | **Sequence coverage (X)** |
| --- | --- | --- | --- | --- | --- |
| Illumina reads | 350bp | 150 | 164.16 | 163.94 | 67.19 |
| Nanopore | - | - | 264.53 | 240.39 | 98.52 |
| Hi-C | 350bp | 150bp | 265.95 | 265.39 | 108.77 |
| Total | - | - | 694.64 | 669.72 | 274.48 |

Note: Estimated genome size of the HL is 2.44 Gb

**Table S2** Repetitive elements statistics in the HL genome

| **Type of repeats** | **Subfamily** | **Number of elements** | **Length occupied (bp)** | **Percentage in the genome (%)** |
| --- | --- | --- | --- | --- |
| SINEs |  | 1,036,614 | 168,398,186 | 6.93 |
|  | ALUs | 137 | 17,991 | 0.00 |
|  | MIRs | 240,994 | 30,209,186 | 1.24 |
| LINEs |  | 1,574,164 | 551,216,960 | 22.68 |
|  | LINE1 | 1,345,857 | 498,112,197 | 20.49 |
|  | LINE2 | 182,599 | 45,341,112 | 1.87 |
|  | L3/CR1 | 19,653 | 4,108,655 | 0.17 |
| LTR elements |  | 381,040 | 128,187,498 | 5.27 |
|  | ERVL | 91,816 | 40,056,970 | 1.65 |
|  | ERVL-MaLRs | 148,105 | 49,224,398 | 2.03 |
|  | ERV_classI | 69,869 | 29,737,709 | 1.22 |
|  | ERV_classII | 6,320 | 1,086,886 | 0.04 |
| DNA elements |  | 492,543 | 78,353,352 | 3.22 |
|  | hAT-Charlie | 179,029 | 33,959,361 | 1.40 |
|  | TcMar-Tigger | 60,512 | 17,764,482 | 0.73 |
| Unclassified |  | 125,154 | 20,016,668 | 0.82 |

Note: DNA: DNA transposons; LINE: Long Interspersed Nuclear Elements; SINE: Short Interspersed Nuclear Elements; LTR: Long Terminal Repeated Elements

**Table S3** The summary of perfect microsatellites detected in the HL genome

| **Type** | **Counts** | **Length (bp)** | **Percent (%)** | **Average Length (bp)** | **Relative Abundance (loci/Mb)** | **Relative Density (bp/Mb)** |
| --- | --- | --- | --- | --- | --- | --- |
| Mono- | 494352 | 7935965 | 37.35 | 16.05 | 203.39 | 3265.11 |
| Di- | 518799 | 12561090 | 39.19 | 24.21 | 213.45 | 5168.03 |
| Tri- | 58490 | 1316070 | 4.42 | 22.5 | 24.06 | 541.47 |
| Tetra- | 193811 | 5155148 | 14.64 | 26.6 | 79.74 | 2120.99 |
| Penta- | 44212 | 1412725 | 3.34 | 31.95 | 18.19 | 581.24 |
| Hexa- | 14057 | 504180 | 1.06 | 35.87 | 5.78 | 207.44 |

**Table S4** Summary statistics of functionally annotated protein-coding genes

| **Type** | **Number** | **Per cent (%)** |
| --- | --- | --- |
| SwissProt | 18,971 | 99.22 |
| TrEMBL | 19,078 | 99.78 |
| KEGG | 12,359 | 64.64 |
| GO | 18,299 | 95.71 |
| InterProsScan | 18,978 | 99.78 |
| Total Annotated Genes | 19,102 | 99.91 |
| Predicted Genes | 19,120 | - |


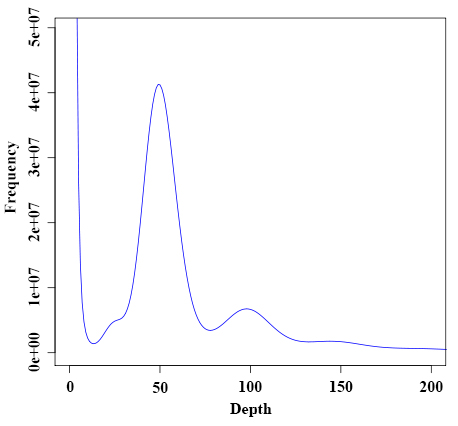


**F_IG_. S1.** 17-mer estimate of the HL genome size. The x-axis is depth (X), the y-axis is the proportion which represents the frequency at that depth divided by the total frequency of all coverage depths.


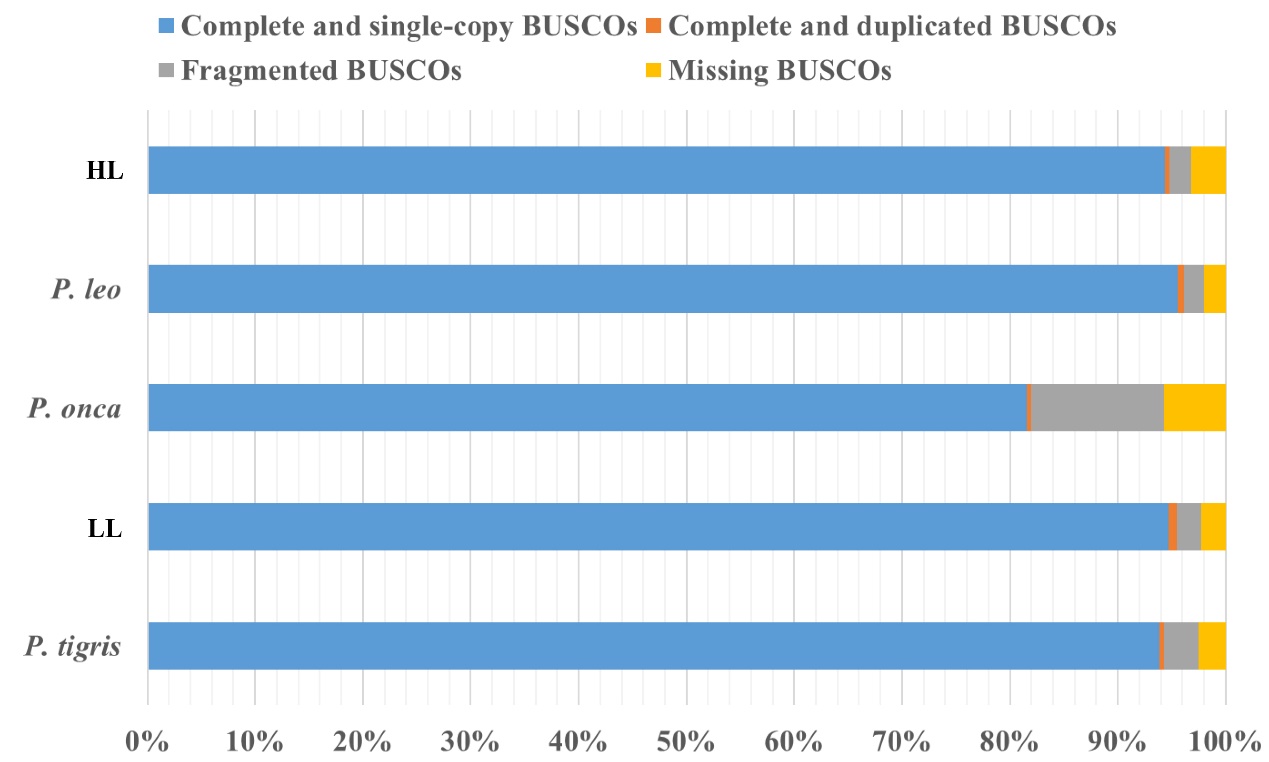


**F_IG_. S2.** Comparison of genome completeness of *Panthera* species.
