# Supplementary figures and images for: Chromosome-level Genome Assembly of the High-altitude Leopard (Panthera pardus) Sheds Light on Its Environmental Adaptation
Source: Genome Biol Evol. 2022 Aug 17;14(9):evac128. doi: 10.1093/gbe/evac128 (PMC9452791; doi:10.1093/gbe/evac128)

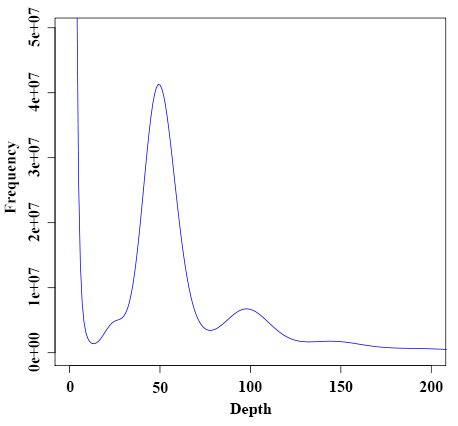

Supplement: evac128_Supplementary_Data [file evac128_supplementary_data.zip › Figure S1.jpg]

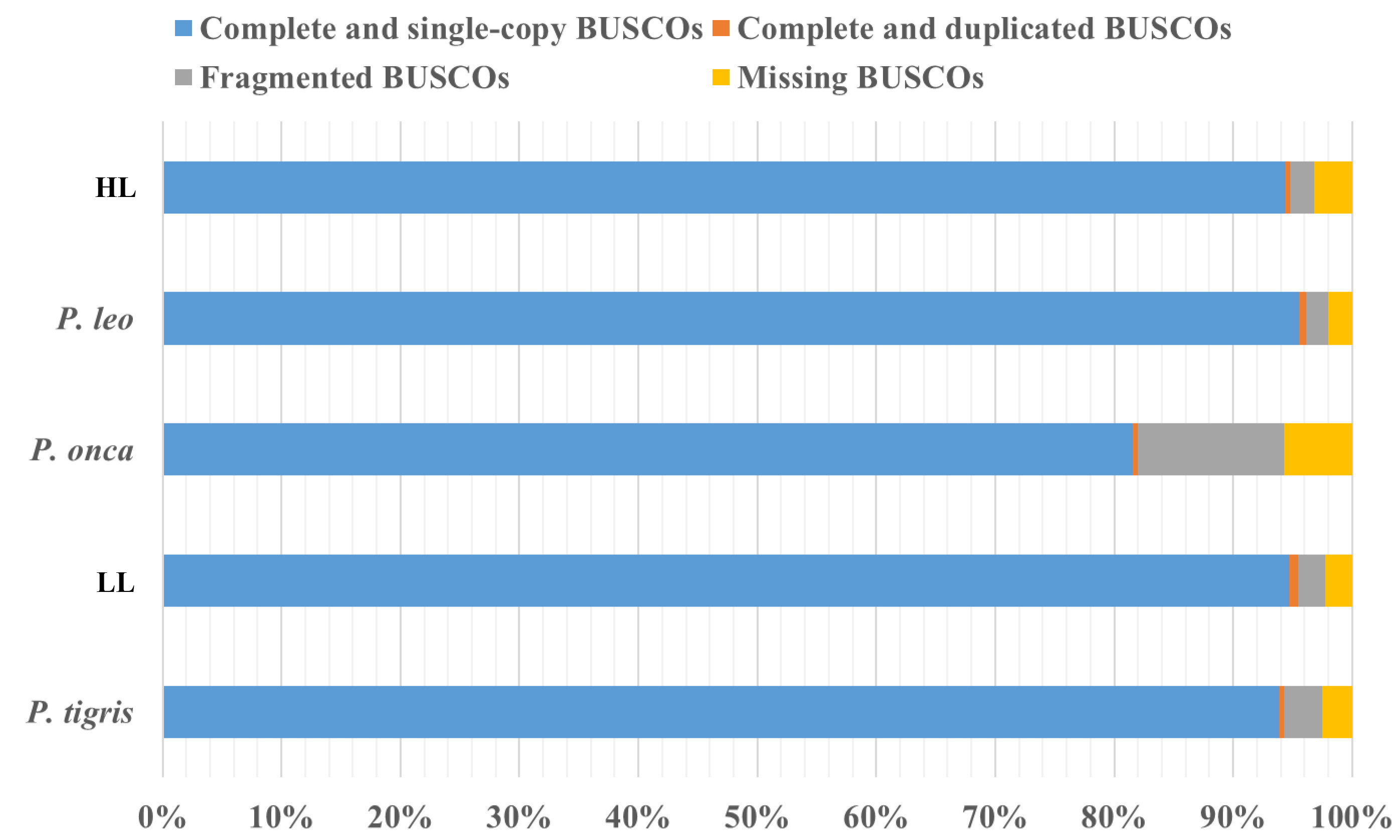

Supplement: evac128_Supplementary_Data [file evac128_supplementary_data.zip › Figure S2.jpg]
